# Predictive factors and adverse perinatal outcomes associated with maternal smoking status

**DOI:** 10.1038/s41598-024-53813-7

**Published:** 2024-02-10

**Authors:** Shereen Hamadneh, Jehan Hamadneh, Esraa Alhenawi, Ruba Abu Khurma, Abdelazim G. Hussien

**Affiliations:** 1 Department of Maternal and Child Health, Faculty of Nursing, Al Al-Bayt University, Mafraq, Jordan; 2 Department of Obstetrics and Gynaecology, Faculty of Medicine, Jordan University of Science and Technology, Irbid, Jordan; 3 Faculty of Information Technology, Zarqa University, Zarqa, Jordan; 4 MEU Research Unit, Faculty of Information Technology, Middle East University, Amman, Jordan; 5 Applied Science Research Center, Applied Science Private University, 11931, Amman, Jordan; 6 Department of Computer and Information Science, Linköping University, Linköping, Sweden; 7 Faculty of Science, Fayoum University, Faiyum, Egypt

**Keywords:** Tobacco, Nulliparous smoking, Risk factors, Cesarean, Applied mathematics, Computer science, Information technology, Scientific data

## Abstract

To identify risk factors for smoking among pregnant women, and adverse perinatal outcomes among pregnant women. A case–control study of singleton full-term pregnant women who gave birth at a university hospital in Jordan in June 2020. Pregnant women were divided into three groups according to their smoking status, active, passive, and non-smokers. They were interviewed using a semi-structured questionnaire that included demographic data, current pregnancy history, and neonatal outcomes. Low-level maternal education, unemployment, secondary antenatal care, and having a smoking husband were identified as risk factors for smoke exposure among pregnant women. The risk for cesarean section was ninefold higher in nulliparous smoking women. Women with low family income, those who did not receive information about the hazards of smoking, unemployed passive smoking women, and multiparty raised the risk of neonatal intensive care unit admission among active smoking women. This risk increased in active and passive women with lower levels of education, and inactive smoking women with low family income by 25 times compared to women with a higher level of education. Smoking is associated with adverse perinatal outcomes. Appropriate preventive strategies should address modifiable risk factors for smoking during pregnancy.

## Introduction

Smoking is associated with many adverse perinatal outcomes and could be controlled and modified by universal efforts^1^. Smoking is associated with congenital malformations^2^, fetal growth restriction^3^, respiratory distress^4^, low birth weight^5^, length and head circumference^6,7^, and premature labour^8,9^. While passive smoking seems to be no less detrimental as it is associated with premature labour too^10,11^, and gestational hypertension^4^.

Furthermore, an independent risk factor for fetal growth limitation is regarded to be paternal smoking^12^, and stillbirth irrespective of maternal smoking status^13^. Higher parental education and monthly family income were protective factors for preterm delivery^8^.

Middle Eastern nations often use tobacco, and Jordan has a high rate of smokers among pregnant mothers^14,15^. The overall smoking rate in Jordan is 40.90%, with a male smoking rate of 59.00% and female smoking rate of 22.80%. Jordan is sixth among world countries and first among the Middle Eastern countries in relation to smoking^16^.

In low- and middle-income nations, smoking is still very common, both actively and passively^17–19^. The question of risk factors for active or passive smoking among pregnant women remains controversial. Younger, less educated pregnant women are more likely to become passive smokers^17,20,21^, where the level of prenatal care had no significant effect^22^. On the other hand, some studies showed that higher level of maternal education, shift work, and unemployment were predictors for smoking during pregnancy^23^. Some studies suggest that low level of maternal education is a very relevant risk factor for smoking among pregnant women^17,24^.

The World “No Tobacco Day (WNTD) 2021 campaign” and "Commitment to quit smoking" amid the COVID-19 pandemic is supported by the Jordanian Ministry of Health (MOH, Jordan 2021). The purpose of this study was to investigate Jordanian women's risk factors for smoking and perinatal outcomes.

### Study objectives

1. Exploring the most common risk factors for smoking among pregnant women.
2. Investigating risk factors for maternal and neonatal outcomes based on maternal smoking status.
3. Examining risk factors for admission to the NICU in relation to maternal smoking status.
4. Analyzing the risk factors associated with cesarean section based on maternal smoking.

## Methods

### Study design and setting

A prospective case–control study was conducted in June 2020 at the King Abdullah University hospital (KAUH), a tertiary referral hospital connected to the Jordan University of Science and Technology in the north of Jordan. This hospital was chosen because it is a major and large hospital in Jordan, serving a substantial number of patients in the northern region of the Kingdom. It provides services for pregnant women and outpatient clinic visits. The presence of a computerized system in the hospital and medical laboratories facilitates the conduct of the study and reaching the target sample. Additionally, there are suitable rooms available to ensure complete privacy and comfort for patients.

### Target population

Full-term singleton pregnant women with an alive fetus, the absence of chronic medical illnesses, pregnancy difficulties, gestational hypertension, and diabetes mellitus were the inclusion criteria for the study. Participants had to agree and complete of the study questionnaire.

### Data collection

Pregnant women who met the inclusion criteria participated in a face-to-face survey, and laboratory and neonatal department records were also made available. A centralized computer database was used to store and analyze the data. The 180 women who were a part of the study were all interviewed using a semi-structured questionnaire that the researchers designed depend on previous studies and it were tested and retested for validity^15,25^. Data were collected from women attending the hospital's gynecology clinics over a period of three months (Please see supplementary materials). Women were invited to participate in the study by a research assistant, and after approval, the form is filled out in a private room while waiting for her appointment at the women’s clinic to maintain privacy and confidentiality. The women's consent was obtained to collect samples, and they were collected in the hospital laboratory by specialists. The examination coincided with requests for regular examinations of pregnant women to monitor the pregnancy by an obstetrician and gynecologist, in accordance with the approved conditions for conducting human research. A minimum sufficient population sample, with a confidence level of 90% and a margin of error of 5%, was determined to be 176. The population was selected using a stratified sampling method. According to the smoking status, questionnaires were divided participants into three groups: Group I current active smokers, Group II of women who were passive smokers, and Group III of women who were neither current passive smokers nor active smokers.

Data collected were included; the independent study variables maternal age, education level, employment status, family income, husband smoking habits, maternal and medical history. The questioner also investigate the awareness of the impacts of smoking were. Dependent factors included perinatal and neonatal outcomes, gestational age at birth, delivery method, birth weight, length, head circumference, Apgar score at one and five minutes, and admission to neonatal intensive care unit. The questionnaire on smoking status categories as [current smoker, non-smoker, and/or expose to Second Hand Smoke (SHS)]. Evaluating smoking status (number of cigarettes smoked per day), and exposure to ambient smoke (type and duration). In addition, we used to validate self-reported smoking status using stem cotinine test. Cotinine is the best biomarker for tracking exposure among non-smokers (passive smoking). Accordingly, this indicator reflects cotinine concentrations in the blood. Active smokers always have serum cotinine levels higher than 10 nanograms per milliliter (ng/ml), while non-smokers exposed to typical levels of passive smoking have serum Concentrations less than one ng/ml. After severe exposure to passive smoking, for non-smokers serum cotinine levels can range between 1 and 10 ng/ml (CDC, 2015, 2017). Therefore, serum cotinine levels were evaluated as [levels of < 10 ng/ml, levels of 10–30 ng/ml, > 30 ng/ml]. All participants were divided into three groups according to their smoking status: group 1, current active smokers; Group 2, women who are currently exposed to passive smoking; the third group, non-smoking women, is neither active nor passive.

### Ethical considerations

The University Review Committee approved this study for Research on Humans. Study participants signed informed consent after providing adequate information. Everyone who participated had the option to withdraw at any moment. All information collected was kept private and confidential. Data were collected accordance with Declaration of Helsinki^26^.

The study was approved by the Institutional Human Research Board (IRB) at Jordan University of Science and Technology, and King Abdullah university hospital. [22/139].

Participation was voluntary and participants could opt out at any time; Signing the consent form indicates consent to participate in the study. The consent form excluded the possibility of undue deception, undue influence or intimidation, and was only signed after potential persons had been adequately informed. Their decision to participate will not affect the doctor-patient relationship or any other benefits to which they are entitled. Personal information about the subjects will never be disclosed, and the data collected will be kept confidential. Furthermore, we can confirm that all experiments were performed in accordance with relevant guidelines and regulations.

### Data analysis

The IBM SPSS 25 application program was used to conduct the statistical analysis (SPSS: An IBM Company, New York, USA). The Shapiro–Wilk W-criterion was used to assess the nature of the data distribution. In accordance with the distributional type of the feature, different statistical analysis procedures were employed. A mean value with a 95% confidence interval was used to convey quantitative data together with its central tendency and dispersion. Bivariate logistic regression analysis determined associations between pregnant women’s characteristics and adverse perinatal outcomes depending on maternal smoking status. To calculate the proportionate rate of differences between the study and control groups, odds ratios (OR) and 95% confidence intervals (CI) were used. If the CI for the OR included 1.0, then the differences between groups as insignificant. If values of CI were more than 1.0, then the studied trait was considered a risk factor. A p-value of 0.05 was used to define statistical significance for all two-sided statistical tests.

## Results

### Characteristic of the studied groups

A total of 180 pregnant women participated in the study, with 60 women in each of the active smoking, passive smoking, and non-smoking groups. Table 1 displays the characteristics of the study participants.

**Table 1:** Characteristic of the study group.

Characteristic	Group I active smoking women (n = 60)		Group II passive smoking women (n = 60)		Group III (control) non-smoking women, (n = 60)	
	Absolute number (%)	95% confidence interval	Absolute number (%)	95% confidence interval	Absolute number (%)	95% confidence interval
Maternal age, Mean ± 95% CI	30.23 ± 1.47		28.85 ± 1.50		31.10 ± 1.34	
Marital status (married)	60 (100)	93.98–100.0	60 (100)	93.98–100	60 (100)	93.98–100
Maternal education						
Secondary school	28 (46.67)	34.63–59.11	6 (10.0)	4.66–20.15	2 (3.33)	0.92–11.36
College	4 (6.67)	2.62–15.93	7 (11.67)	5.77–22.18	2 (3.33)	0.92–11.36
Bachelor’s degree	26 (43.33)	31.57–55.89	31 (51.67)	39.31–63.83	44 (73.33)	60.99–82.86
Postgraduate degree	2 (3.33)	0.92–11.36	16 (26.67)	17.14–39.01	12 (20.0)	11.83–31.78
Working status (work)	18 (30.0)	19.9–42.51	27 (45.0)	33.09–57.51	32 (53.33)	40.89–65.37
Family monthly income, Jordanian dinars						
≤ 350	14 (23.33)	14.44–35.43	2 (3.33)	0.92–11.36	6 (10.0)	4.66–20.15
351–500	24 (40.0)	28.57–52.63	26 (43.33)	31.57–55.89	22 (36.67)	25.62–49.32
501–1000	16 (26.67)	17.14–39.01	25 (41.67)	30.07–54.28	22 (36.67)	25.62–49.32
> 1000	4 (6.67)	2.62–15.93	7 (11.67)	5.77–22.18	10 (16.67)	9.32–28.04
Nulliparous	8 (13.33)	6.91–24.16	26 (43.33)	31.57–55.89	16 (26.67)	17.14–39.01
Antenatal care (more than 3 visits)	50 (83.33)	71.96–90.68	50 (83.33)	71.96–90.68	58 (96.67)	88.64–99.08

### Risk factors for smoking among Jordanian women

The study found that smoking during pregnancy was more likely in women with low levels of maternal education, such as those who only completed secondary school. Compared to women who were employed, those without jobs smoked more frequently. Lack of regular antenatal care during pregnancy increased the risk of smoking, both active and passive. Women whose husbands were smokers had a 38-fold higher risk to be also smokers, as well as to be passive smokers. Maternal age, family monthly income, parity, and awareness about the hazards of smoking did not affect the risk for active and passive smoking among pregnant women. The details of the risk factors for smoking among participants are represented in Table 2.

**Table 2:** Risk factors of smoking among Jordanian pregnant women.

Characteristic	Active smoking women (n = 60)		Passive smoking women (n = 60)	
	OR [95% CI]	P-value, p	OR [95% CI]	P-value, p
Maternal age ≤ 20 years	2.80 [0.83–9.49]	0.153	4.21 [0.46–38.87]	0.364
Maternal education				
Secondary school	25.38 [5.67–113.51]	< 0.0001	3.22 [0.62–16.66]	0.272
Bachelor’s degree and more	0.06 [0.02–0.19]	< 0.0001	0.26 [0.08–0.85]	0.034
Maternal unemployment status	2.67 [1.26–5.64]	0.016	1.39 [0.68–2.86]	0.465
Family monthly income ≤ 350 JD	2.74 [0.97–7.70]	0.085	0.31 [0.06–1.60]	0.272
Multiparity	2.36 [0.92–6.04]	0.109	0.48 [0.22–1.02]	0.084
Antenatal care (less than 3 visits)	5.80 [1.21–27.73]	0.029	5.8 [1.21–27.73]	0.029
Smoking husband	38.00 [10.58–136.55]	< 0.0001	236[29.62–1880.2]	< 0.0001
Lack of knowledge about smoking hazards	2.80 [0.83–9.49]	0.153	0.48 [0.09–2.74]	0.679

### Risk factors of adverse pregnancy outcomes and maternal smoking status

Nulliparous smoking women had a ninefold higher risk of having a cesarean section (CS), while non-smoking or passive smoking women did not have this risk. Low family monthly income increased the risk of CS among women of Group I, while unemployment increased it more than sixfold in women of Group II. Factors that increase the need for CS are represented in Table 3.

**Table 3:** Risk factors for Cesarean section according to maternal smoking status.

Studied factors	Group III (control) non-smoking women, n = 60		Group I active smoking women, n = 60		Group II passive smoking women, n = 60	
	OR [95% CI]	P-value, p	OR [95% CI]	P-value, p	OR [95% CI]	P-value, p
Maternal age > 35 years	2.92 [0.89–9.53]	0.085	0.52 [0.13–2.09]	0.491	2.57[0.47–13.95]	0.446
Maternal education ≤ 12 years	0.64 [0.05–7.47]	0.999	2.31 [0.82–6.56]	0.128	0.75 [0.09–5.71]	0.999
Unemployed maternal status	0.56 [0.19–1.57]	0.305	1.67 [0.55–5.07]	0.409	6.25[2.02–19.32]	0.002
Family monthly income ≤ 350 JD	2.91 [0.49–17.29]	0.388	7.8 [1.56–38.88]	0.006	1.56[0.13–18.23]	0.999
Nulliparity	0.72 [0.22–2.33]	0.769	9.0 [1.05–77.29]	0.029	2.53 [0.87–7.39]	0.117
Perinatal following up (> 3 visits)	0.36 [0.03–4.25]	0.574	6.0 [1.15–31.23]	0.035	7.11[1.36–37.16]	0.015
Smoking husband	1.32 [0.08–22.15]	0.999	1.22 [0.42–3.58]	0.787	2.25 [0.56–8.99]	0.305
Absence of information about smoking hazards during perinatal visits	0.72 [0.22–2.33]	0.769	4.38[1.45–13.29]	0.009	0.81 [0.26–2.5]	0.777

The current study showed that maternal age at delivery significantly increases the risk of NICU admission in all three groups of women. Multiparty raised this risk more than 10 times in Group I women, whereas in Group II nulliparity was a risk factor for NICU admission, while multiparity was a protective factor. Women who smoked, both actively and passively, and had lower levels of education were at much higher risk for NICU admission. Additionally, low family monthly income negatively affected the risk of NICU admission women of Group I. Risk factors for NICU admission are represented in Table 4.

**Table 4:** Risk factors for NICU admission according to maternal smoking status.

Characteristic	Group III (control) non-smoking women, n = 60		Group I active smoking women, n = 60		Group II passive smoking women, n = 60	
	OR [95% CI]	P-value	OR [95% CI]	P-value	OR [95% CI]	P-value
Maternal age > 35 years	15.77 [1.69–147.52]	0.008	19.55 [2.07–184.87]	0.004	17.5 [1.86–164.53]	0.006
Maternal education ≤ 12 years	2.41 [0.14–40.83]	0.999	186.33 [17.83–1947.37]	< 0.0001	17.5 [1.86–164.53]	0.006
Family monthly income ≤ 350 Jordanian Dinars	1.19 [0.19–7.15]	0.999	4.11 [1.15–14.73]	0.038	0.80 [0.08–8.29]	0.999
Parity > 1	1.9 [0.38–5.12]	0.755	10.38 [1.26–85.79]	0.025	0.29 [0.09–0.95]	0.046
Smoking husband	2.41 [0.14–40.83]	0.999	1.71 [0.47–6.21]	0.541	1.71 [0.32–9.05]	0.709

In all groups of pregnant women, this study’s variables did not affect the rates of CS, preterm delivery, low birth weight, length, head and chest circumferences, low Apgar scores at 1st and 5th minutes, and NICU admissions.

## Discussion

One of the avoidable risk factors for poor prenatal outcomes is smoking, which the world's health care systems should address^1^.

This study found that the strongest predictive factor for active and passive smoking among pregnant women was having a smoking husband. Paternal smoking increases the risk of maternal smoking by 38 times and second-hand smoking by 236 times. The second important risk factor was low-level maternal education, secondary school or less, which raised the chance to be a smoker by 25 times compared to women with a higher level of education. Having a bachelor’s degree or higher reduced the chances for being an active or passive smoker. These findings are supported by other studies^17,21,24^.

Being unemployed had more than the two-fold risk of being a smoker compared to those women who worked during pregnancy. This finding is comparable to the findings of other studies where being unemployed increased the risk of being a pregnant smoker^21,23^.

Additionally, this study revealed that poor antenatal care increased the risk of both active and passive smoking. This is probably due to the less responsible approach to childbirth, or due to getting less information about the dangers of smoking during pregnancy. In contrast, Garg and Mora-Pinzon found that the level of prenatal care did not affect maternal smoking status^22^.

Women who smoke are more likely to have a CS for non-reassuring fetal status^27^. However, this study found that there are additional risk factors that increase the incidence of CS in women who were active or second-hand smokers. These additional risk factors for needing a CS were nulliparity, low family income, poor antenatal care and lack of adequate knowledge about the hazards of smoking during pregnancy.

Li and colleagues^27^ showed that smoking significantly increases the prevalence of NICU admission^27^. This study found, in addition, that maternal age significantly increases the risk of NICU admission in active and passive smokers, as well as non-smoking women. Furthermore, multiparty increased the risk of NICU admission in actively smoking women, those with lower level of education, and low income. Among passive smoking women, the factor that significantly increased the rate of NICU admission was lower levels of education.

In all participant groups, other research variables had no significant effect on the probability of CS, preterm delivery, low birth weight, length, head, or chest circumferences, low Apgar score at 1 or 5 min, or NICU admission. These results are particularly supported by another study which revealed that there is no correlation between smoking status and Apgar score at 5 min^6^. The authors are not aware of other studies with similar methodology that aimed at identifying risk factors for adverse perinatal outcomes in relation to maternal smoking status.

This study supports the previous research that indicates that smoking among pregnant women, both passive and active, is an issue that needs the implementation of intervention for the prevention of risks associated with tobacco use^14^. There is still a high percentage of passive and active smoking among pregnant women in Jordan ^15^. In response, the Jordan Ministry of Health issued plans to combat smoking and indicated that Jordan was able to provide the capabilities for citizens to quit smoking through the implementation of the necessary programs and policies, and the opening of cessation clinics^28^, in concordance with the World No Tobacco Day (WNTD) 2021 campaign and "Commitment to quit smoking" amid the COVID-19 pandemic.

### Strengths and limitations

This study could act as a baseline for other larger studies and could help stakeholders and decision-makers to implement smoking prevention strategies. It excluded high-risk pregnant women, discussed a limited number of perinatal outcomes, and did not consider pregnancy complications such as gestational diabetes, gestational hypertension, fetal abnormalities, and placental abruption, which could be related to smoking.

This study included cotinine measurement. This is not considered a "gold standard" in pregnant women, especially for passive smoking verifying^20^, but may give some indication of the extent of the exposure to smoke. Further research on this issue is mandated (Supplementary information).

## Conclusions and recommendations

This study demonstrated that smoking during pregnancy—both active and passive—increases the rate of cesarean section (CS) and neonatal intensive care unit admission, especially in women with additional risk factors such as maternal age, nulliparity, low level of education, low family income, maternal unemployment, and lack of knowledge about the hazards of smoking during pregnancy. Furthermore, the study discovered several indicators that determine whether pregnant women may smoke actively or passively that, in addition to the risk factors for higher CS and NICU admissions, include paternal smoking status, and deficient of antenatal care. Health care workers should consider these factors during evaluation encounters.

### Supplementary Information

Supplementary Information 1.

Supplementary Information 2.

## Data Availability

Data is available based on reasonable request from the corresponding author.

## References

[1] Committee opinion no. 721 summary: Smoking cessation during pregnancy. *Obstet. Gynecol* **130**(4), 1. 10.1097/AOG.0000000000002348(2017). 10.1097/AOG.0000000000002348 28937568

[2] Raitio A, Heiskanen S, Syvänen J, Leinonen MK, Kemppainen T, Löyttyniemi E, Helenius I (2023). Maternal risk factors for congenital vertebral anomalies: A population-based study. J. Bone Jt. Surg..

[3] Zhang Q, Zhang ZC, He XY, Liu ZM, Wei GH, Liu X (2022). Maternal smoking during pregnancy and the risk of congenital urogenital malformations: A systematic review and meta-analysis. Front. Pediatrics.

[4] Adibelli D, Kirca N (2020). The relationship between gestational active and passive smoking and early postpartum complications. J. Matern. Fetal. Neonatal. Med..

[5] Huang SH, Weng KP, Huang SM, Liou HH, Wang CC, Ou SF, Lin CC, Chien KJ, Lin CC, Wu MT (2017). The effects of maternal smoking exposure during pregnancy on postnatal outcomes: A cross-sectional study. J. Chin. Med. Assoc. JCMA.

[6] Kharkova OA, Grjibovski AM, Krettek A, Nieboer E, Odland JØ (2017). Effect of smoking behavior before and during pregnancy on selected birth outcomes among singleton full-term pregnancy: A Murmansk county birth registry study. Int. J. Environ. Res. Public Health.

[7] Rumrich I, Vähäkangas K, Viluksela M, Gissler M, de Ruyter H, Hänninen O (2020). Effects of maternal smoking on body size and proportions at birth: a register-based cohort study of 1.4 million births. BMJ Open.

[8] Ferguson KK, Rosario Z, McElrath TF, Vélez Vega C, Cordero JF, Alshawabkeh A, Meeker JD (2019). Demographic risk factors for adverse birth outcomes in Puerto Rico in the PROTECT cohort. PLoS ONE.

[9] Liu B, Xu G, Sun Y, Qiu X, Ryckman KK, Yu Y, Snetselaar LG, Bao W (2020). Maternal cigarette smoking before and during pregnancy and the risk of preterm birth: A dose-response analysis of 25 million mother-infant pairs. PLoS Med..

[10] Hoyt AT, Canfield MA, Romitti PA, Botto LD, Anderka MT, Krikov SV, Feldkamp ML (2018). Does maternal exposure to secondhand tobacco smoke during pregnancy increase the risk for preterm or small-for-gestational age birth?. Matern. Child Health J..

[11] Rang NN, Hien TQ, Chanh TQ, Thuyen TK (2020). Preterm birth and secondhand smoking during pregnancy: A case-control study from Vietnam. PLoS ONE.

[12] Chen MM, Chiu CH, Yuan CP, Liao YC, Guo SE (2020). Influence of environmental tobacco smoke and air pollution on fetal growth: A prospective study. Int. J. Environ. Res. Public Health.

[13] Qu Y, Chen S, Pan H, Zhu H, Yan C, Zhang S, Jiang Y (2020). Exposure to tobacco smoke and stillbirth: A national prospective cohort study in rural China. J. Epidemiol. Community Health.

[14] Hamadneh S, Kassab M, Eaton A, Wilkinson A, Creedy DK, Laher I (2020). Sudden unexpected infant death. Handbook of Healthcare in the Arab World.

[15] Hamadneh J, Hamadneh S, Amarin Z, Al-Beitawi S (2021). Knowledge, attitude and smoking patterns among pregnant women: A Jordanian perspective. Ann. Glob. Health.

[16] World Population Review. *Smoking Rates by Country 2021*. https://worldpopulationreview.com/country-rankings/smoking-rates-by-country.

[17] Madureira J, Camelo A, Silva AI, Reis AT, Esteves F, Ribeiro AI, Teixeira JP, Costa C (2020). The importance of socioeconomic position in smoking, cessation and environmental tobacco smoke exposure during pregnancy. Sci. Rep..

[18] Mahmoodabad S, Karimiankakolaki Z, Kazemi A, Mohammadi NK, Fallahzadeh H (2019). Exposure to secondhand smoke in Iranian pregnant women at home and the related factors. Tobacco Prev. Cessation.

[19] Reece S, Morgan C, Parascandola M, Siddiqi K (2019). Secondhand smoke exposure during pregnancy: A cross-sectional analysis of data from Demographic and Health Survey from 30 low-income and middle-income countries. Tobacco Control.

[20] Hikita N, Haruna M, Matsuzaki M, Sasagawa E, Murata M, Oidovsuren O, Yura A (2017). Prevalence and risk factors of secondhand smoke (SHS) exposure among pregnant women in Mongolia. Sci. Rep..

[21] Kondracki AJ (2019). Prevalence and patterns of cigarette smoking before and during early and late pregnancy according to maternal characteristics: The first national data based on the 2003 birth certificate revision, United States, 2016. Reprod. Health.

[22] Garg S, Mora-Pinzon MC (2019). Trends and risk factors of secondhand smoke exposure in nonsmoker pregnant women in Wisconsin, 2011–2016. WMJ.

[23] de Wolff MG, Backhausen MG, Iversen ML, Bendix JM, Rom AL, Hegaard HK (2019). Prevalence and predictors of maternal smoking before and during pregnancy in a regional Danish population: A cross-sectional study. Reprod. Health.

[24] Härkönen J, Lindberg M, Karlsson L, Karlsson H, Scheinin NM (2018). Education is the strongest socio-economic predictor of smoking in pregnancy. Addiction.

[25] Chaaya M, Jabbour S, El-Roueiheb Z, Chemaitelly H (2004). (2004) Knowledge, attitudes, and practices of argileh (water pipe or hubble-bubble) and cigarette smoking among pregnant women in Lebanon. Addict. Behav..

[26] Goodyear MD, Krleza-Jeric K, Lemmens T (2007). The declaration of Helsinki. BMJ..

[27] Li R, Lodge J, Flatley C, Kumar S (2019). The burden of adverse obstetric and perinatal outcomes from maternal smoking in an Australian cohort. Austral. New Zeal. J. Obstet. Gynaecol..

[28] The Jordanian World Health Organization (2021). http://www.moh.gov.jo/DetailsPage/MOH_EN/NewsDetailsAR.aspx?ID=741. Accessed 31 May 2021.

